# Study on the Strength of the Brake Pad of a Freight Wagon under Uneven Loading in Operation

**DOI:** 10.3390/s24020463

**Published:** 2024-01-11

**Authors:** Sergii Panchenko, Juraj Gerlici, Alyona Lovska, Vasyl Ravlyuk, Ján Dižo, Jozef Harušinec

**Affiliations:** 1 Department of Automation and Computer Telecontrol of Trains, Ukrainian State University of Railway Transport, Feuerbach Square 7, 61050 Kharkiv, Ukraine; panchenko074@ukr.net; 2 Department of Transport and Handling Machines, University of Zilina in Zilina, Univerzitná 1, 010 26 Žilina, Slovakia; juraj.gerlici@fstroj.uniza.sk (J.G.); alyona.lovska@fstroj.uniza.sk (A.L.); jozef.harusinec@fstroj.uniza.sk (J.H.); 3 Department of Wagon Engineering and Product Quality, Ukrainian State University of Railway Transport, Feuerbach Square 7, 61050 Kharkiv, Ukraine; ravvg@ukr.net

**Keywords:** transport mechanics, brake pad, method for the pad calculation, uneven loading on the pad, pad stress, train safety

## Abstract

The paper highlights the results of determining the strength of the brake pad of a freight wagon under uneven loading in operation. The main reasons for the uneven loading on the pad have been found. A mathematical tool for determining the strength of the pad unevenly loaded has been proposed. In the study, the pad is considered to be a rod system loaded with concentrated forces and bending moments. Sensors have been used in order to detect the load state of the brake pads. These sensors have been defined in the simulation software, and they have been placed on the working surface of the pad in the area of its interaction with the wheel. The operation of these sensors was simulated in the simulation software package. The results of the calculation have shown that the stresses in the pad are about 21.1 MPa; thus, they exceed the permissible values by 29%. Therefore, considering the uneven loading of the pad in operation, the strength of the pad is not ensured. To test the obtained results, the strength of the pad was determined using the finite element method. The Coulomb criterion was used for the calculation. It was found that the maximum stresses in the pad were about 19 MPa. These stresses were 21% higher than permissible values and occurred in the back of the pad. The study has proven that the uneven loading on the brake pad in operation can cause their destruction during braking. This may also cause traffic accidents with freight trains during their movement. The results of this study will contribute to the theoretical developments and recommendations aimed at improving the brake system of a freight wagon and rail traffic safety. It is considered that the tensometric sensors will be applied in future experimental tests for comparison and verification of the achieved results from the simulation computations.

## 1. Introduction

In order to ensure timely transportation, the rolling stock, along with other technical means, must guarantee the safety of railway traffic and failure-free operation, as well as ensure railway capacity.

**Figure 1:**
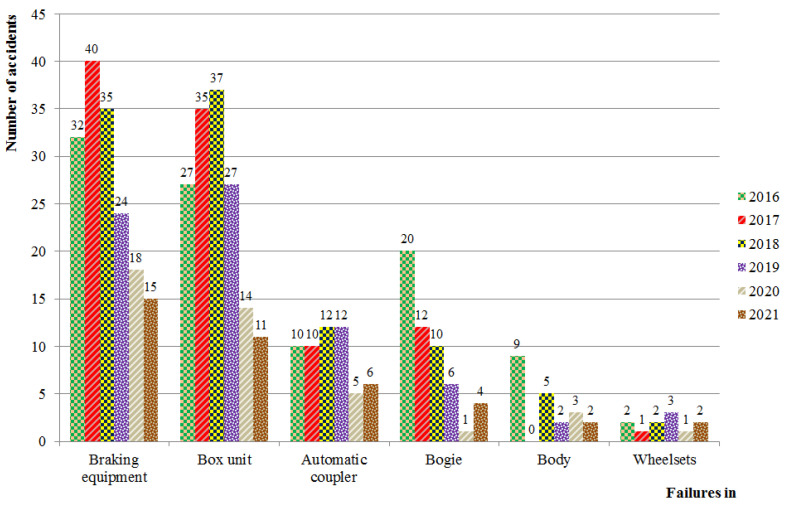
Comparative bar graph of accidents in the wagon industry by types of failures in freight wagon units.

By analyzing a number of rail traffic accidents in the example of the wagon fleet of Ukrainian Railways (Ukrzaliznysia), it is possible to see that most of them have been caused by failures in the brake equipment. At the same time, the number of traffic accidents by year of the calculation period has been constantly decreasing (Figure 1). The main reason for this is the reduction in the number of freight wagons in Ukrzaliznytsia. Over the last few years, the number of wagons repaired at car repair enterprises (depots, factories) has been gradually decreasing. Thus, the rate of reducing the number of traffic accidents in relation to the number of repaired wagons over previous years is 0.22%. This indicates that the reduction in the number of accidents in the wagon industry mainly depends on the number of repaired wagons, and the problems of train traffic safety are yet to be solved [1].

Based on the analysis (Figure 1), it has been found that the unsatisfactory operation of the brake equipment over the period from 2016 to 2021 was the cause of 40 accidents in 2017; it was the largest number of accidents in the wagon industry in the mentioned period. In this regard, it has been decided to provide a more thorough quantitative assessment of failures related merely to the brake equipment of freight wagons that can cause accidents on the railways of Ukraine.

Based on the obtained results of observations carried out under operating conditions of 1520 mm gauge freight cars, it was found that one of the most vulnerable elements of the brake system is the pads. At the moment, the 1520 mm gauge freight wagons use composite brake pads, which have the following chemical composition: 20% of rubber, 47.5% of baride, 15% of soot, 2.5% of vulcanizing agent, and 15% of other additives.

It is important to note that one of the most common defects of such pads is their abnormal wear. The formation and development of abnormal pad wear depends on the design and condition of the brake system of the car bogie. When the freight wagon reaches a mileage of about 80,000 km, its worn-out pad, which still has 39% of the working mass on average, should be replaced (the design service life is 160,000 km); this requires extra operating costs that can be avoided by upgrading the brake leverage system of freight wagons.

**Figure 2:**
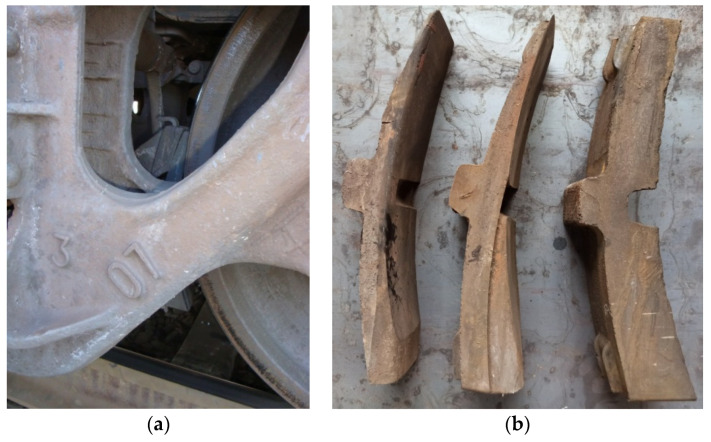
Unevenly worn composite brake pads that are unsuitable for further use: (**a**) identified during maintenance of wagons and should be replaced; (**b**) removed from wagons during repair.

The analysis conducted has made it possible to identify the specific wear of brake pads only on their upper parts; this local friction abrasion appears not during braking but when the wagon operates at the mode of traction or braking (Figure 2). It has been found that almost 90% of the brake pads in each freight train are tilted, pressed against the rolling surfaces of the wheels, and sustain harmful friction when the train moves without braking at the modes of traction or run-out. This significantly reduces the service life of the pad (by almost 50%), as well as the service life of wagon wheelsets, thus causing high-temperature damage on the rolling surfaces. Such friction increases the main resistance to the train movement on which the locomotive wastes up to 19.7% of energy resources depending on the traction type. The working friction area of the pad is reduced, which negatively affects the braking efficiency and requires the replacement of the pad even with a large residual working mass.

In this regard, it is important to study the causes of uneven loads of pads in operation, determine their strength, and create measures aimed at eliminating their uneven wear in operation.

## 2. Analysis of Recent Research and Publications

Issues related to the safety of freight rolling stock are quite relevant and important. Study [2] presents an analysis of the stress state of the composite brake pad using SolidWorks software version 2015 (Dassault Systèmes SOLIDWORKS Corp., Waltham, MA, USA). The pad is made of innovative friction material based on modified alkyl benzene resin, which makes it possible to considerably improve the friction coefficient of brake pads used for rolling stock. In [3], a dynamic model of the shoe brake of industrial rolling stock is presented in the form of a design diagram in which a flat contact is implemented. Some limitations of the model that can greatly simplify analytical calculations are provided. The developed dynamic model of the shoe brake allows controlling the friction force by magnitude and speed function depending on the parameters of the dynamic brake processes. At the same time, the authors of works [2,3] did not take into account the uneven loading of the pad during braking.

The authors of study [4] considered a method for temperature stabilization when the brake pad contacted the wheel and proposed some structural improvements. Temperature distribution has been detected by means of thermal sensors. Among them are inserts in the brake pad made of a special material that can expand during braking. The diameter and depth of holes that help reduce wear are substantiated. However, the authors did not take into account the design features of the brake leverage system of freight wagon bogies, which can cause abnormal pad wear and negatively affect braking efficiency and train safety. However, the use of innovative brake pads in freight wagon bogies can only significantly increase the operating costs and will not provide the desired result regarding reliable brake operation.

Study [5] highlights the results of tests on wagon rolling stock in order to assess the braking efficiency. The study was carried out to find the type of structural uncertainty during the identification of braking conditions by changing the friction coefficients of the brake pads that interact with the wheel and depend on speed, basic resistivity to train movement, type of braking, etc. However, it should be noted that the braking process is affected not only by the coefficient of friction of the brake pad, which depends on the material but also by many other factors, for example, the load of the pad during its interaction with the wheel. This research has been carried out on a test device, which included special rotational-velocity sensors, whose signals have been processed by means of a computer.

Study [6] deals with the impact of braking on the longitudinal and transverse wagon dynamics. The graphical dependences of the sensitivity of dynamic processes in wagons to the braking force are provided. Also highlighted are issues related to rail defects and how exactly they affect the change in the coefficient of friction of the tribotechnical pair “brake pad-wheel”. The research required the consideration of the sensors for detection of the braking/traction torque effect for examination of the lateral and longitudinal wagon dynamics. However, the authors did not consider the surface defects of the wheels arising due to the prolonged interaction with heavily worn pads, which plays a very important role in assessing the braking and dynamic processes occurring in rolling stock.

The study [7] presents the results of research aimed at eliminating the angular movements of the brake cylinder rod in conjunction with the horizontal lever, which has a kinetostatic interconnection during the maximum travel of the rod. The authors developed measures to improve the brake cylinder by changing its design, which prevents the displacement of the rod at its maximum travel. The proposed structural improvements in the brake cylinder are substantiated by strength calculations. However, the issue of how uneven wear of the pad would affect the travel value of the brake cylinder rod was not studied.

The work [8] is aimed at elucidating the operation features of cast iron and composite pads of rolling stock. The authors presented the negative characteristics of composite brake pads causing high-temperature defects on the wheels, which require higher operating and transportation costs.

In the freight wagon, the load is transmitted to the brake leverage of the bogie from the brake cylinder located on the wagon frame. During braking, the rods and levers move due to the kinetostatic connections, and, as a result, each pad gets a force load and becomes pressed to the wheel. However, due to the dynamic processes during movement, when the wheelsets interact with isolated irregularities of the track, the balance of the brake leverage is disturbed, and the pads wear out unevenly, which is the main cause of their over-normative wear [9]. To avoid it, constructive changes are made in triangle systems and brake leverage elements, which can prevent uneven wear of pads; the features of this scientific approach are provided in the publication [10]. Different types of brake leverage systems with devices that ensure uniform wear of pads and their advantages and drawbacks are also provided. The study outlines a scientific approach based on kinetostatic analysis, which allows considering the dynamic components in the unsprung part of the wagon bogie. According to the results of the study, the causes of uneven wear of composite brake pads were identified, and ways to eliminate them were provided. Despite the positive solutions presented in the study, it should be noted that the stress state of unevenly worn brake pads was not calculated.

Currently, the main normative and technical document regulating the reliable operation of brakes of rolling stock used for the 1520 mm gauge is [11]. However, it should be noted that this document does not provide any recommendations regarding uneven wear of composite brake pads in freight wagons, which adversely affects the components and units of the wagons, as well as the condition of the track when the freight train moves on the rail section. This may be explained by the fact that this type of wear has not been sufficiently studied regarding the bogie model 18–100 used for the 1520 mm gauge.

Studies [12,13] are aimed at analyzing the operation of tribotechnical units and substantiating the introduction of promising materials in their design. Due to such solutions, the train speed and the load on the wagon axle increase significantly, the life of tribotechnical parts (composite brake pads, linings, and bushings used in kinetostatic units) increases, the operation of the brake system of rolling stock improves, etc. However, there are a number of problems related to the uneven wear of composite brake pads in freight trains, which requires considerable attention. Thus, the problems related to the abnormal wear of the elements of the tribotechnical unit “brake pad-wheel” of freight wagons are actually quite relevant [14,15].

Studies [16,17] deal with brake pads operating in extremely difficult conditions, and special requirements regarding the materials for them are described. These materials must have high strength and wear resistance to avoid cracking and fracture and sufficient hardness to ensure minimal wear of the wheel during braking [18]. This can be achieved by changing the structure of the material the pads are made of. But, at the same time, the tribotechnical properties of brake pads must include a significant coefficient of friction in order to urgently stop the vehicle in case of emergencies while minimizing its braking distance.

By analyzing literary sources [2,3,4,5,6,7,8,9,10,11,12,13,14,15,16,17,18], it has been possible to conclude that issues of uneven wear of composite brake pads of freight wagons used on the 1520 mm gauge are quite relevant and require further research and development.

## 3. Purpose and Tasks of the Research

The purpose of this study is to create a scientific approach to determining the strength of the brake pad of a freight wagon under uneven loading in operation. To achieve this purpose, the following objectives are defined:

- To determine the causes of the uneven loading on the brake pad in operation;
- To offer a mathematical tool for determining the strength of the brake pad unevenly loaded;
- To investigate the stress distribution fields in the brake pad loaded unevenly.

## 4. Determination of Causes of the Uneven Loading in Operation

To regulate the train speed and stop, it is necessary to perform stepped or full-service braking, during which the brake pad contacts with the wheel and friction wear develops. According to the results of observation of composite brake pads removed during the repair of freight wagons used on the 1520 mm track gauge, it has been found that a considerable number of the pads have uneven wear. The main reasons for such wear are as follows:

- Uneven distribution of the specific pressure on the brake pad due to the shift of the response of the wheel to the pad relative to the symmetry axis toward the leading end of the pad;
- The pad touches the wheel with the upper end during the train movement due to the imperfect design of the brake lever. This is explained by the fact that the axis passing through the center of the hole in the brake strut does not coincide with the axis of the pendulum suspensions that hold the composite brake pads;
- During the operation of freight wagons, wear is generated in the kinetostatic connections of the brake leverage, which causes a significant increase in clearances, which, in turn, causes an uneven distribution of specific pressures on the pad and, as a result, uneven wear of the pads;
- Failures in the device for the parallel retraction of brake shoes, which result in constant contact of the upper part of the pad and the wheel during the train movement with the brakes released.

**Figure 3:**
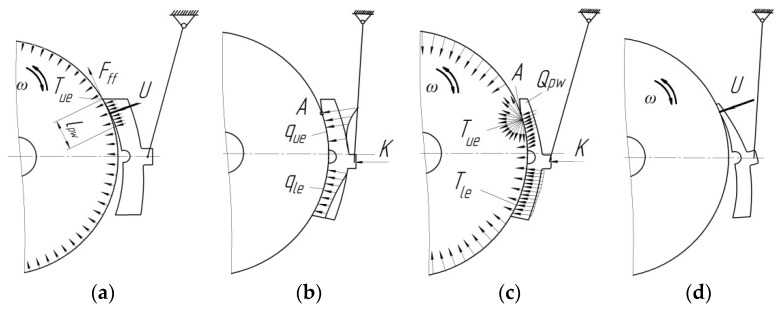
Stages of development of the uneven wear on the composite brake pad of freight wagons: (**a**) new pad leans with its upper end against the surface of the wheel without braking; (**b**) uneven distribution of the brake contact pressures *q_ue_* and *q_le_* from the pad to the wheel at the uneven wear; (**c**) braking accompanied by an increase in the heat dissipation *T_ue_* and *T_le_* due to the uneven wear of the pad; (**d**) heavily worn brake pad with large residual working mass; must be replaced.

The study of cases of abnormal wear of brake pads [19,20] indicates that a new pad starts to wear out mainly in its upper part (Figure 3a), and the result is the low reliability of the device for the parallel retraction of brake shoes. When the wagon reaches a mileage of about 3000 km, the wear becomes double and looks like the end wear with significant friction abrasion at the upper end of the pad, which is rather harmful (Figure 3b). The lower working part of the brake pad wears out unevenly and faster near the upper abrasion with a gradual deceleration to the bottom.

Therefore, it should be noted that the upper parts of the brake pad wear out more intensively than the lower ones (Figure 3c). The upper part of the pad *l*_pw_, which is shortened due to abrasion, concentrates a much higher specific pressure *q_ue_* than does the lower part *q_le_*. As a result, the top of the pad wears out more quickly than expected, and it depends on the mileage of the freight wagon; this requires replacing the brake pad with a large residual working mass, especially in the lower part, which could be used effectively during normal operation of the device for the parallel retraction of brake shoes (Figure 3d). During braking, the heat dissipation *T_ue_* increases significantly below the line of plane separation (point A) with a slow deceleration to the brake pad bottom *T_le_* (Figure 3c).

As a rule, the uneven wear of composite brake pads develops due to the low reliability of the brake leverage of the bogie and an imperfect design of the brake beam, where the gravitational force is formed by the weight of the lever transmission parts [19]. Under this force, the pad is tilted and pressed by the slight force *U* against the rolling surface of the wheel, which rotates in a direction that depends on the train’s movement. Therefore, when the wagon mileage increases, an unevenly worn plane marked with the symbol *Q*_pw_ on the pad is formed; it will gradually increase. The specified force with the specific brake pressure *q*_pw_ distributes on it; the friction force *F_ff_* also appears.

## 5. Development of a Mathematical Tool for Determining the Strength of an Unevenly Loaded Brake Pad

### General Provisions and Geometrical Characteristics of the Composite Brake Pad

**Figure 4:**
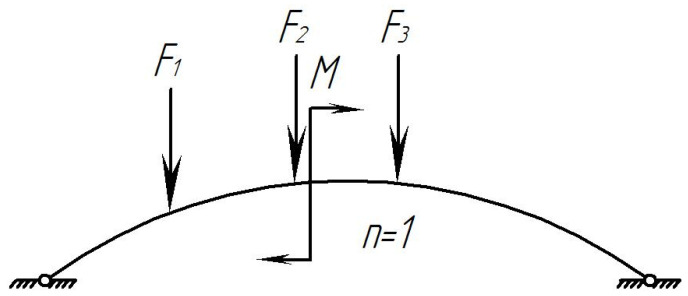
Design diagram of the brake pad: *F* is the force transmitted from the brake shoe to the pad; *M* is the torque moment.

In order to determine the strength of the composite brake pad that is loaded unevenly, a calculation was made. The pad was considered to be a rod system on two supports (Figure 4).

**Figure 5:**
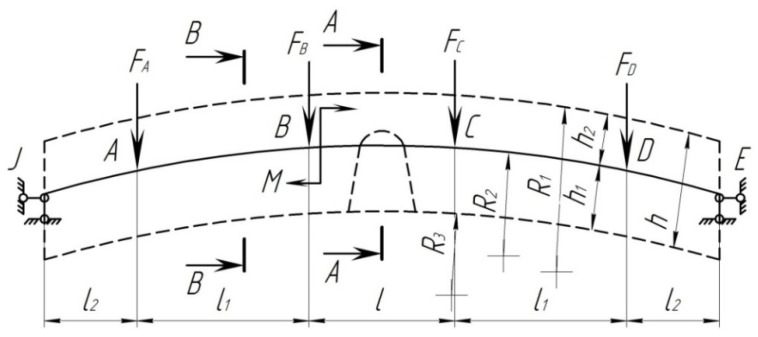
Design diagram of the composite brake pad.

The design diagram includes a curvilinear axis that passes through the center of gravity of the pad cross-section. The position of the center of gravity is determined in Figure 5. In this case, the radius of the neutral axis of the pad is denoted by *R*. Further, this diagram will be referred to as the arch.

Due to the fact that the arch is a curved nonlinear outline, the method of its calculation depends on the curvature, which is determined by the following formula:

(1) $$k=\frac{h}{R},$$

where *h* is the pad height and *R* is the average radius of the pad.

In accordance with [21], it is commonly supposed to distinguish between small and large curvature beams. If *k* < 0.2, then it is a beam of small curvature. In the design diagram, *k* < 0.2 since the beam has a small curvature. For engineering calculations, small curvature beams can be determined by the formulas provided for beams with a straight axis.

However, it should be noted that the effect of arbitrary forces increases in low arches; therefore, on the basis of [21], for a beam with the rise *f* < l/3, the effect of longitudinal forces should not be neglected. *f* is the rise of the curvilinear beam, and *l* is the length of the curvilinear beam (here, it is the length of the pad).

The rise of the curvilinear beam is determined from Figure 4.

(2) $${\left(\frac{l}{2}\right)}^{2}+{\left(R-f\right)}^{2}=R^{2},\mathrm{or}\;f^{2}-2\cdot R\cdot f+\frac{l^{2}}{4}=0.$$

Then,

(3) $$f=\frac{2\cdot R}{2}\pm \frac{1}{2}\cdot \sqrt{4\cdot R^{2}-4\cdot 1\cdot \frac{l^{2}}{4}}.$$

After that, it is necessary to check the following condition:

(4) $$f<\frac{l}{3}.$$

Figure 5 shows the design diagram of the pad, taking into account a uniformly distributed load across the area. During braking, the pad perceives the force transmitted from the shoe, the force of friction, and the temperature effect due to the interaction with the wheel. At the initial stage of calculation, it is assumed that the pad perceives the force acting on it from the shoe and the force of friction.

The direction of the loads acting on the pad coincides with the direction of the pad axis. The neutral axis connecting the centers of gravity of the sections of the pad is taken as the calculation axis. Since most parts have a constant cross-section, it is possible to neglect the center of gravity of cross-section A-A in determining the radius of the pad (Figure 5).

**Figure 6:**
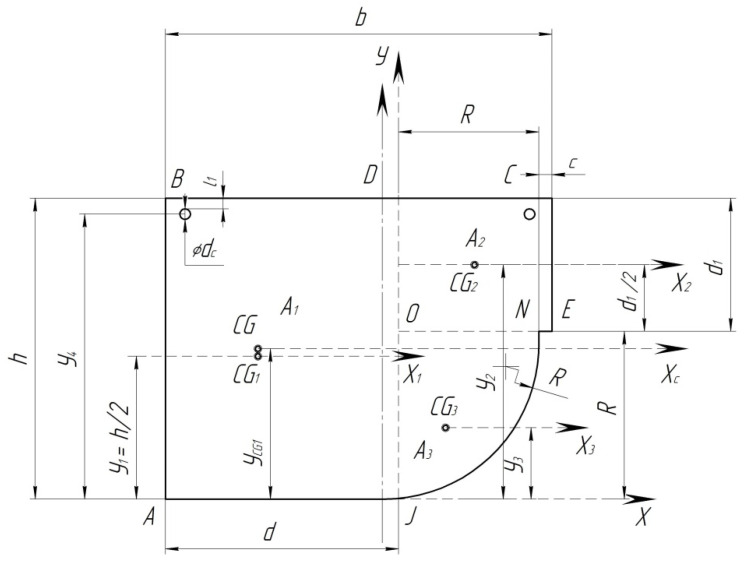
Position of the center of gravity of the composite brake pad.

Determine the position of the center of gravity of section B-B of the pad (Figure 6). To do this, divide the section into elementary figures, the centers of gravity of which are easy to determine.

The position of the center of gravity of the section of the pad is determined by the following formula:

(5) $$y_{CG}=\frac{\sum S_{x}^{i}}{\sum A^{i}},$$

where $\sum S_{x}^{i}$ is the sum of static moments of sectional areas; $\sum A^{i}$ is the sum of the areas that make up the section.

The total area of the sections of the pad is given as two rectangles, *ABCD = A_3_*.

Determine the static moments of the components of the sections relative to the arbitrarily selected axis *X* (Figure 6).

The static moments of the rectangle *ABDJ* relative to the *X*-axis are as follows:

(6) $$S_{1}=A_{1}\cdot y_{1}=d\cdot h\cdot \frac{h}{2}.$$

The static moments of the rectangle *ODCE* relative to the *X*-axis are as follows:

(7) $$S_{2}=A_{2}\cdot y_{2}=R\cdot d_{1}\cdot \left(\frac{d_{1}}{2}+R\right).$$

The area of a quadrant, according to [22], is determined by the following formula:

(8) $$A_{3}=\frac{\pi \cdot d^{2}}{16}=0.196\cdot d^{2}.$$

The position of the center of gravity relative to the axis, according to [22], is determined by the following formula:

(9) $$y_{3}=0.2878\cdot d.$$

The static moments of a quadrant are as follows:

(10) $$S_{3}=A_{3}\cdot y_{3}.$$

In addition, there are two metal rods with a diameter of 4 mm.

(11) $$S_{4}=\frac{\pi \cdot d^{2}}{4}\cdot 2\cdot y_{3}.$$

The total static moments relative to the X-axis will be equal to

(12) $$\sum S=S_{1}+S_{2}+S_{3}+S_{4}.$$

Accordingly, the total sectional area is determined by

(13) $$\sum A=A_{1}+A_{2}+A_{3}+A_{4}.$$

The position of the center of gravity of the entire cross-section relative to the *X*-axis is determined by Formula (3).

The position of the center of gravity of the cross-section can be used to determine the radius of the pad for the design diagram.

In addition, according to Figure 6, determine the axial moment of inertia of the cross-section of pad *I* since it can be used to determine the stiffness of the beam at bending *E*∙*I* [kN/mm^3^] where *E* is the modulus of elasticity of the material, kN/mm^2^; *I* is the axial moment of inertia of the cross-section relative to the main central axis, mm^4^.

Since the pad consists of two materials, steel with *E_st_* = 2∙10^5^ MPa and composite with *E_st_′* = 5∙10^3^ MPa, when determining the axial moments of inertia of the cross-sections, it is necessary to take into account the correction factor, which includes an increase in the stiffness of the metal due to the following composite:

(14) $$K_{1}=\frac{E_{st}}{E_{st}^{\prime }}.$$

Determine the axial moment of inertia of the cross-section relative to the main central axis (Figure 6).

The axial moment of inertia of the rectangle *ABDJ* relative to the axis *X_c_* is

(15) $$I_{1}=I_{X1}+A_{1}\cdot {\left(y_{CG1}-\frac{h}{2}\right)}^{2}.$$

The axial moment of inertia of the rectangle *ODCE* relative to the axis *X_c_* is

(16) $$I_{2}=I_{X2}+A_{2}\cdot {\left(h-\frac{d_{1}}{2}-y_{CG2}\right)}^{2}=\frac{R\cdot d_{1}^{3}}{12}+R\cdot d_{1}\cdot {\left(h-\frac{d_{1}}{2}-y_{CG2}\right)}^{2}.$$

The axial moment of inertia of a quadrant relative to the axis *X_c_*, according to [22], is

(17) $$I_{3}=I_{X3}+A_{3}\cdot {\left(y_{CG3}-y_{3}\right)}^{2}.$$

The axial moment of inertia of two metal rods with a diameter of 4 mm is

(18) $$I_{4}=2\cdot \left[\frac{\pi \cdot d_{c}^{4}}{64}+\frac{\pi \cdot d_{c}^{2}}{4}\cdot {\left(h-3-\frac{d_{2}}{r}-y_{CG}\right)}^{2}\right].$$

The total axial moment of inertia of the cross-section B-B, taking into account the correction factor for steel is

(19) $$I_{B-B}=I_{1}+I_{2}+I_{3}+I_{4}\cdot K_{1}.$$

**Figure 7:**
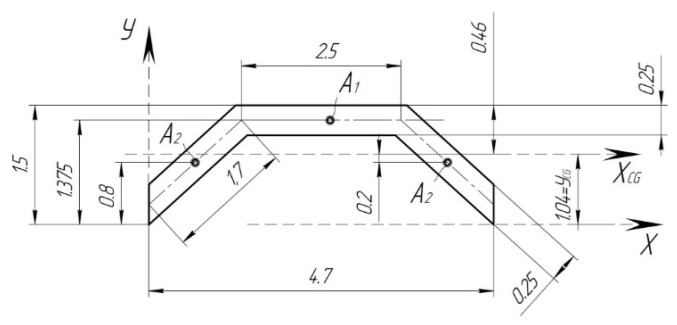
Cross-section of one corrugation crinkle of the composite brake pad.

**Figure 8:**
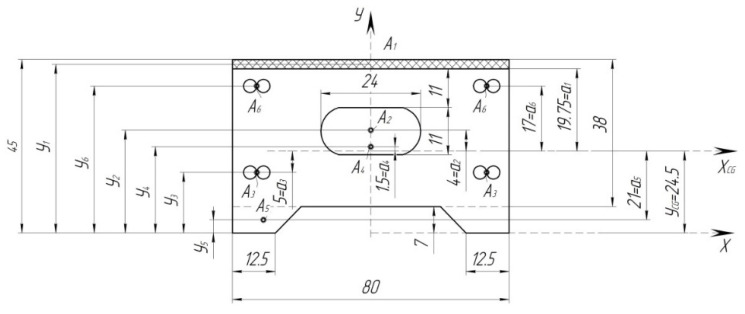
Cross-section A-A of the composite brake pad.

Determine the axial moment of inertia of the pad across A-A (Figure 5). The cross-section obtained from [21] is shown in Figure 7 and Figure 8.

In the cross-section shown in Figure 8, there is a wave of corrugation (Figure 7). Determine the position of the center of gravity of the corrugation section and the axial moment of inertia.

Randomly select the corrugation axis *X* and, with respect to it, determine the static moments of the constituent cross-sections *A*_1_ and *A*_2_ (Figure 7).

Then, the total sectional area will be

(20) $$A=A_{1}+2\cdot A_{2}.$$

Determine the position of the center of gravity of the cross-section relative to the *X*-axis by Formula (5)

(21) $$y_{CG}=\frac{S}{A}.$$

Determine the axial moments of inertia of the constituent sections $I_{X_{CG}}^{A_{1}}$ and $I_{X_{CG}}^{A_{2}}$ relative to the axis *X_CG_*.

The axial moment of inertia of one corrugation relative to its central axis *X_CG_* is equal to

(22) $$I^{W}=I_{X_{CGT}}^{A_{1}}+I_{X_{CG}}^{A_{2}}.$$

The sectional area of one corrugation is equal to

(23) $$A^{W}=A_{1}+2\cdot A_{2}.$$

Hereafter, determine the geometric characteristics of the entire cross-section A-A (Figure 8). In order to determine the position of the center of gravity of the cross-section relative to the arbitrarily selected axis, determine the areas of the constituent cross-sections.

Since the area of one corrugation is determined, and in the cross-section of the pad, there are only 17 corrugations, then the total area of all corrugations will be

(24) $$A_{1}=A^{W}\cdot 17.$$

The static moments relative to the *X*-axis can be determined by the known following formula:

(25) $$S_{i}=A_{i}\cdot y_{i}.$$

The axial moments of inertia relative to the axis *X_CG_* (Figure 8) can be determined as

(26) $$I_{1}=I^{W}+A_{1}\cdot a_{1}^{2}.$$

(27) $$I_{3}=4\cdot \frac{\pi \cdot d^{4}}{64}+A_{3}\cdot a_{3}^{2}.$$

(28) $$I_{4}=\frac{b\cdot h^{3}}{12}+A_{4}\cdot a_{4}^{2}.$$

(29) $$I_{5}=\frac{b\cdot h^{3}}{12}+A_{5}\cdot a_{4}^{2}.$$

(30) $$I_{6}=4\cdot \frac{\pi \cdot d^{4}}{64}+A_{6}\cdot a_{6}^{2}.$$

The next stage of the calculation includes the determination of the axial moment of inertia of the cross-section A-A, taking into account the correction factor for steel rods:

(31) $$\sum I_{A-A}=I_{1}-I_{2}+I_{3}\cdot K.$$

When calculating a statically indeterminate system, it is more convenient to reduce the axial moments of inertia of all cross-sections to the minimum value. Accept the value $I_{A-A}=I_{o}$ , then

(32) $$I_{B-B}=\frac{139.9}{115.18}=1.24\cdot I_{0}.$$

The following is suggested:

- At the moment of braking, the pad has a degree of freedom equal to 1 (Figure 4);
- The coefficient of friction between the pad and the wheel is *ϕ_ff_* = 0.3;
- The pressure from the shoe to the pad is applied as a distributed load, and for calculation, it is replaced by concentrated forces. In braking, it is distributed along the middle of the pad;
- The pressure transmitted from the shoe to the pad is directed vertically, not in the direction of the cross-sections;
- The friction forces, according to [23], are directed in a straight line;
- The pressure from the shoe to the wheel is transmitted in the form of an unevenly distributed load, which increases from the ends to the middle of the bar. It can be determined by placing the pad on the supports located at characteristic points.

**Figure 9:**
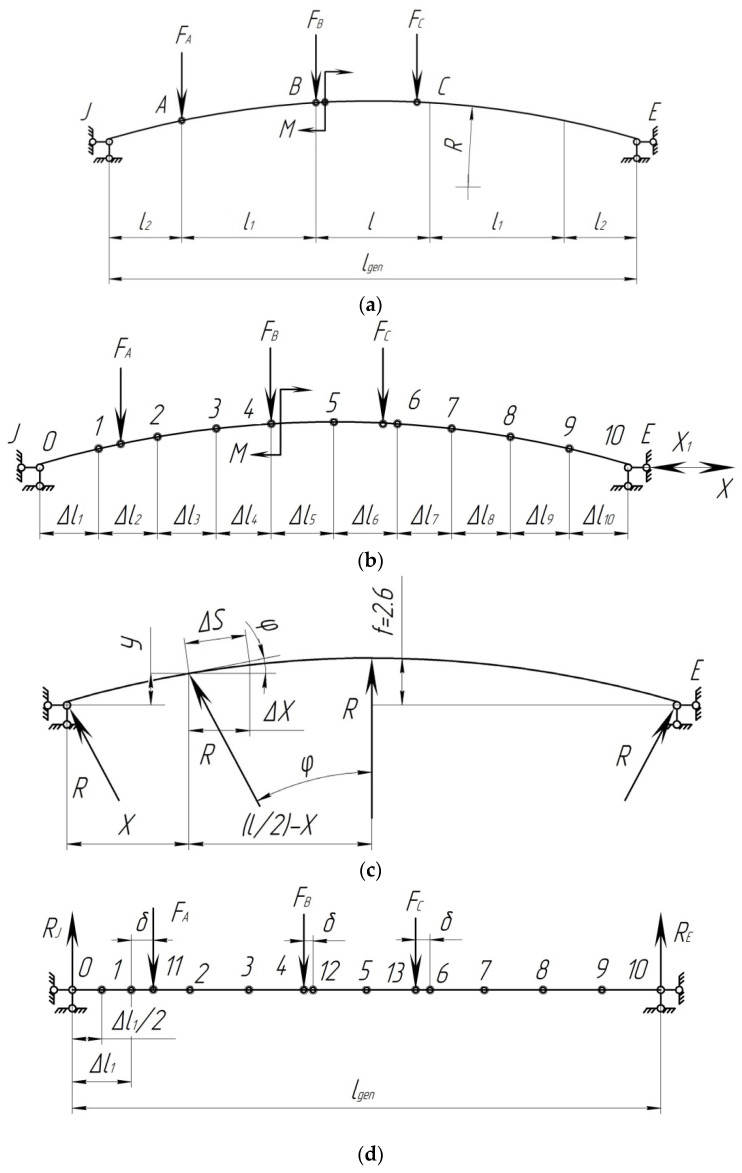
Auxiliary diagrams for determining unknown canonical equations: (**a**) main system; (**b**) main system with the unknown strut *X*_1_; (**c**) auxiliary diagram for determining unknown reactions; (**d**) forces from the external vertical load.

Figure 9a shows the given pad system, and Figure 9b shows the main pad system in the form of a curvilinear beam. The canonical equation of the force method can be written as

(33) $$\delta_{11}\cdot X_{1}+\Delta_{11}=0.$$

Then, the displacements in *δ*_11_ and Δ_11_ are determined using the Mohr formula:

(34) $$\begin{array}{l} \delta_{11}=\int_{0}^{S}\frac{{\bar{M}}_{1}^{2}}{E\cdot I}\cdot dS+\int_{0}^{S}\frac{{\bar{N}}_{1}^{2}}{E\cdot A}\cdot dS,\\ \;\\ \Delta_{11}=\int_{0}^{S}\frac{\bar{M}\cdot M_{f}}{E\cdot I}\cdot dS+\int_{0}^{S}\frac{\bar{N}\cdot N_{f}}{E\cdot A}\cdot dS. \end{array}$$

In these formulas, the integration is performed along the length of the arch axis.

Here, ${\bar{M}}_{1}$ and ${\bar{N}}_{1}$ are the bending moment and the longitudinal force from *X*_1_ = 1;

A is the cross-sectional area of the pad.

Under the action of force *X*_1_ = 1 (Figure 9b), internal forces occur in the cross-sections of the arch:

(35) $${\bar{M}}_{1}=-y;\;{\bar{N}}_{1}=-\cos\varphi .$$

In Formulas (33) and (34), there is no lateral force, as noted above. In low arches (arches of small curvature), the influence of the lateral force can be neglected.

The forces from the external vertical load can be expressed through the beam forces (Figure 9d):

(36) $$M_{f}=M_{0}\;\;:\;\;N_{f}=-\sin\varphi .$$

Denote by $I_{0}=I_{A-A}$ the moment of inertia in the cross-section A-A, then, taking into account Expression (34), the formulas for determining *δ*_11_ and Δ_11_ can be obtained in this form:

(37) $$\begin{array}{l} EI_{0}\delta_{11}=\int_{0}^{S}y^{2}\frac{I_{0}}{I}dS+\int_{0}^{S}\cos^{2}\varphi \frac{I_{0}}{A}dS,\\ \;\\ EI_{0}\Delta_{1f}=-\int_{0}^{S}yM_{0}\frac{I_{0}}{I}dS+\int_{0}^{S}Q_{0}\sin\varphi \cos\varphi \frac{I_{0}}{I}dS, \end{array}$$

where $I_{0}=I_{A-A}$ ; *I*—in the cross-section of the *i*-th point;

A is the cross-sectional area in the *i*-th point.

Analytical calculation of integrals (37) in this case is difficult; therefore, it is often replaced by numerical calculation. Therein, the arch axis is divided into arbitrarily small sections of the length ΔX , and the forces of all magnitudes for the middle of each section are determined in accordance with [24]:

(38) $$\begin{array}{l} E\cdot I_{0}\cdot \delta_{11}=\sum y^{2}\cdot \frac{I_{0}}{I}\cdot \Delta S+\sum \cos^{2}\varphi \cdot \frac{I_{0}}{A}\cdot \Delta S,\\ \;\\ E\cdot I_{0}\cdot \Delta_{1f}=-\sum y\cdot M_{0}\cdot \frac{I_{0}}{I}\cdot \Delta S+\sum Q_{0}\cdot \sin\varphi \cdot \cos\varphi \cdot \frac{I_{0}}{I}\cdot \Delta S. \end{array}$$

After determining *δ*_11_ and Δ_11_, $X=-\frac{\Delta_{1f}}{\delta_{11}}$ is found and the internal force of the given arch is determined by the following formulas:

(39) $$\begin{array}{l} M=M_{0}-y\cdot X_{1};\\ Q=Q_{0}\cdot \cos\varphi -\sin\varphi \cdot X_{1};\\ N=-Q_{0}\cdot \sin\varphi -\cos\varphi \cdot X_{1}, \end{array}$$

where $M_{0}$ and $Q_{0}$ are the beam bending moments and the lateral force.

In order to solve the problem, use Formula (38) and neglect the lateral deformations. According to Figure 9c, find the necessary geometric and trigonometric relations:

(40) $$\begin{array}{l} \Delta_{3}=\frac{\Delta X}{\cos\varphi };\;y=\sqrt{r_{2}-{\left(\frac{l}{2}-X\right)}^{2}}-r+f;\\ \;\\ \sin\varphi =\frac{\frac{l}{2}-X}{r};\;\cos\varphi =\frac{\sqrt{r_{2}-{\left(\frac{l}{2}-X\right)}^{2}}}{r}. \end{array}$$

Divide the curvilinear beam into 10 equal parts so that $\Delta X=\frac{l_{gen}}{10}$ (Figure 9b). Points 1, 2, 3… are the midpoints of the sections since different external loads act on the beam in the form of concentrated forces and bending moment, and for this case, consider two diagrams separately. The first diagram is in the form of a curvilinear beam with external concentrated forces, which follows from Figure 9a by the remote bending moment. Figure 9b shows the main system with the unknown strut *X*_1_ and without bending moment. The forces from the external vertical load can be expressed through the beam forces (Figure 9d).

**Figure 10:**
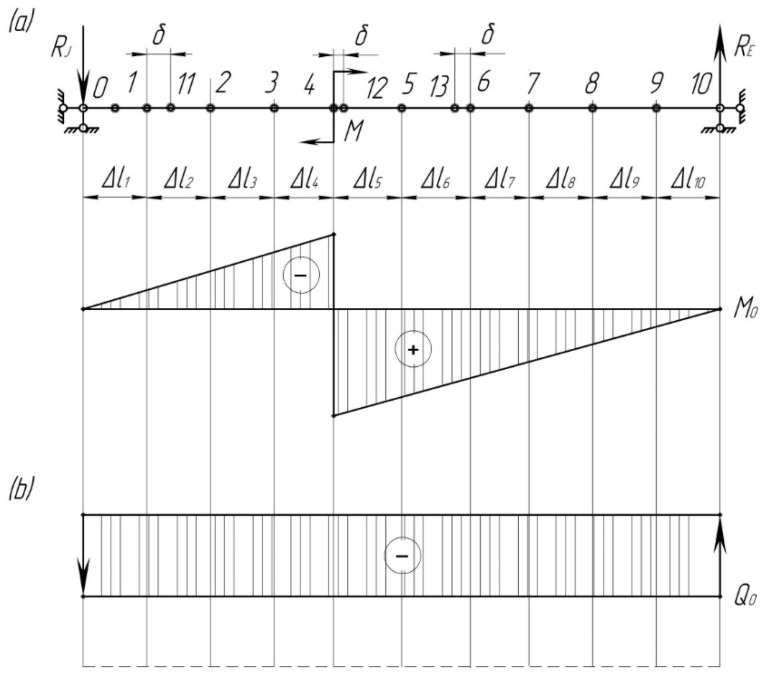
Beam forces from the concentrated moment: (**a**) bending moment diagram; (**b**) beam lateral force diagram.

The next step includes the determination of the forces that act on the pad from the bending moment. To do this, construct diagrams of the beam bending moment $M_{0}$ and the beam lateral force $Q_{0}$ (Figure 10).

It should be noted that the calculation did not include the impact of lateral forces; however, the diagrams of the lateral forces were included.

**Figure 11:**
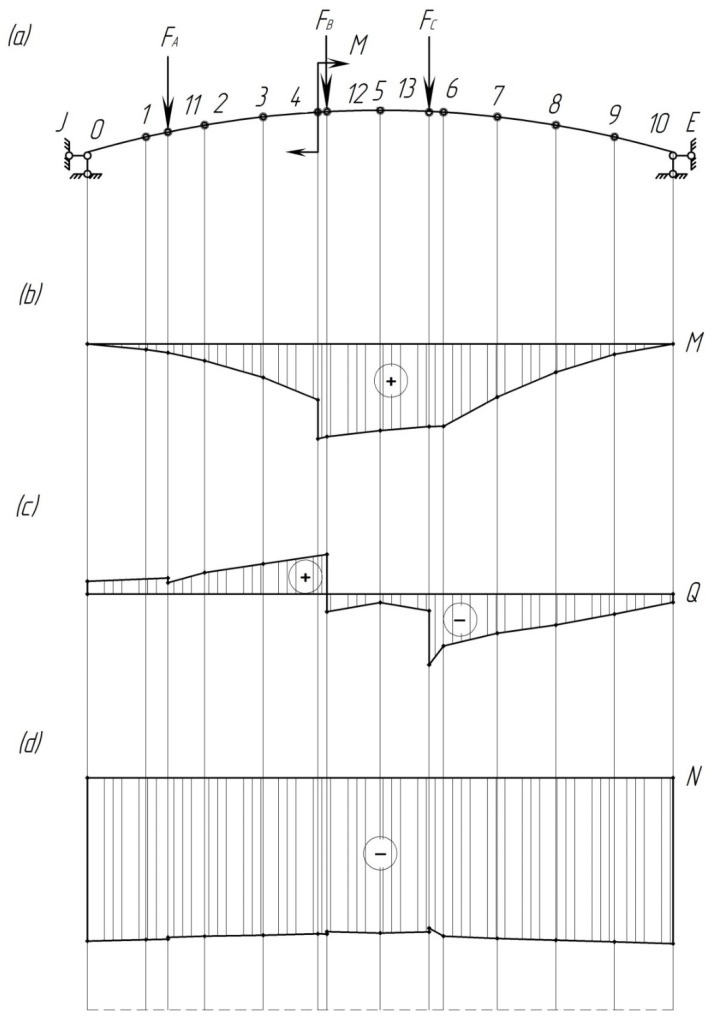
Diagrams of bending moments, lateral and longitudinal forces acting on the pad: (**a**) design diagram; (**b**) bending moment diagram; (**c**) lateral force diagram; (**d**) longitudinal force diagram.

From the diagrams shown in Figure 11, it follows that the dangerous cross-section is point 12, in which the axial resistance moment is equal to

(41) $$W_{A-A}=\frac{I_{A-A}}{y_{CG}}.$$

The stress in this cross-section is equal to

(42) $$\sigma_{A-A}=\frac{M_{12}}{W_{A-A}}.$$

For the cross-section,

(43) $$W_{B-B}=\frac{I_{B-B}}{y_{CG}}.$$

The dangerous point for these cross-sections is point 6 $M_{6}$ .

(44) $$\sigma_{B-B}=\frac{M_{6}}{W_{B-B}}.$$

By taking into account the calculation carried out at *F* = 20.55 kN and *M* = 68.8 kN∙m, it has been found that the stresses that occur in the pad are 21.1 MPa. The resulting stress value exceeds the permissible value, which, in accordance with [25], is taken equal to 15 MPa.

The calculation makes it possible to conclude that if, under the action of static forces on the pad, its strength is not ensured, then there is no need for thermal calculation.

The proposed mathematical tool can be used to optimize the geometric parameters of the pad using the resistance moment and taking into account the values of the stresses acting on it. It is important to note that this calculation method was developed for the first time because, previously, no attention was paid to the study of the strength of brake pads, taking into account their uneven loading. Due to the fact that there is currently a problem of uneven wear of brake pads, which certainly affects the safety of trains, the authors proposed this calculation method.

The disadvantage of this method is that it cannot be used to determine the stress distribution fields across the pad area since it is considered a rod system. Therefore, a further stage of research includes the calculation of the pad using the finite element method.

## 6. Investigation of Stress Distribution Fields in the Brake Pad Unevenly Loaded

In order to determine the strength of the composite pad, taking into account the uneven loading on it, a simulation calculation was made. For this purpose, the finite element method was implemented in SolidWorks Simulation.

**Figure 12:**
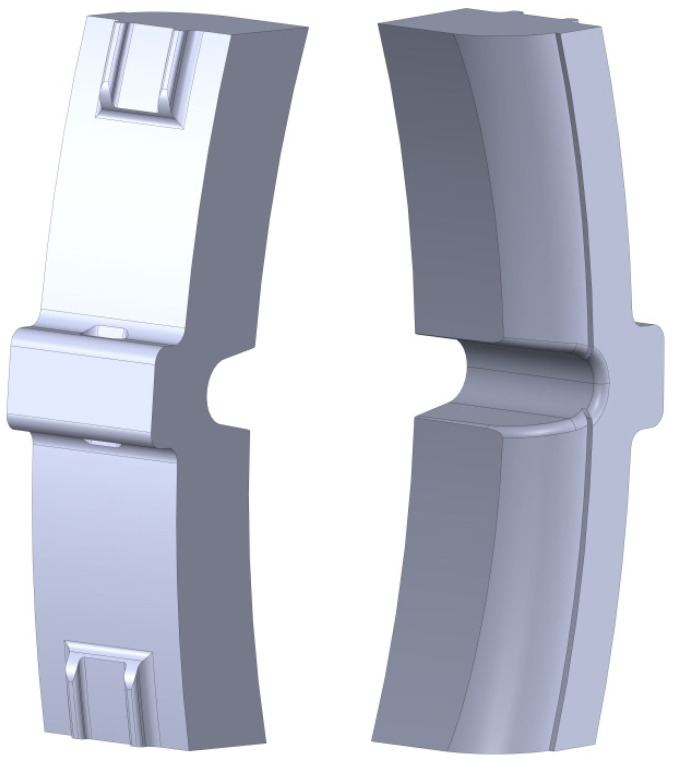
Spatial model of the composite brake pad.

A spatial model of the brake pad was created in SolidWorks (Figure 12).

Tetrahedral elements were used to create the finite element model [26,27]. Their optimal number was determined by the graphic-analytical method [28,29,30,31,32]. The FEM of the pad had 5424 nodes and 24,421 elements with a maximum size of 15 mm and a minimum size of 3 mm. The sensors of the load have been installed on the working surface of the pad in the wheel/pad interaction area in the simulation software. These sensors have been used to detect the load of the calculated pad under the prescribed operational load. The operation principle of the sensors is similar to that of real tensometric sensors during a real test.

The pad was fixed at its back in the contact area to the shoe. The pad was made of a composite material with linear elastic orthotropic properties. The main characteristics of this material are summarized in Table 1.

At the same time, the compressive strength of the material was assumed 15 MPa, and the tensile strength was close to zero.

**Figure 13:**
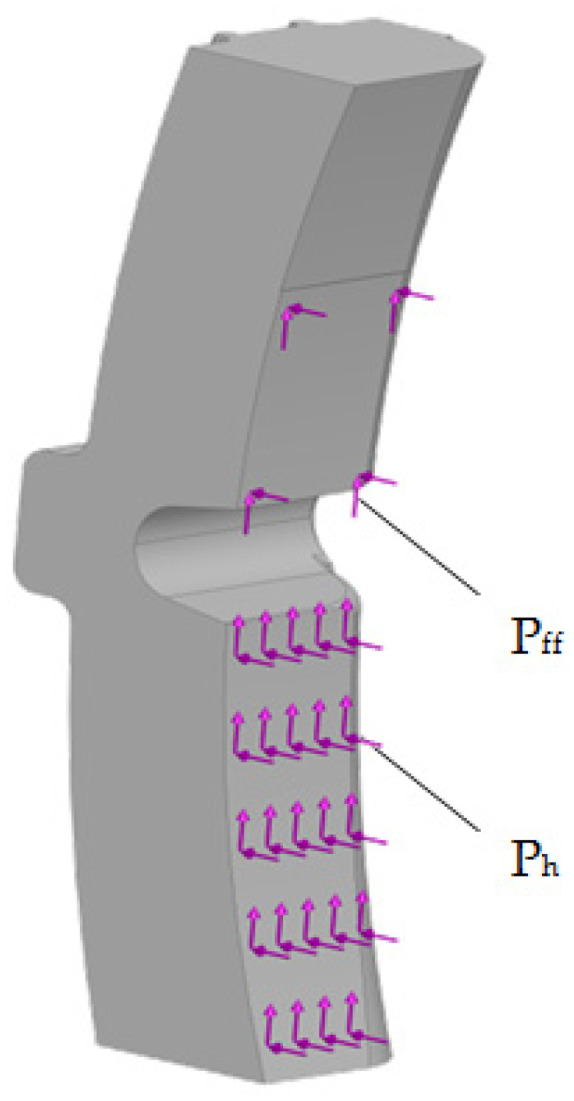
Design diagram of the pad.

The design diagram of the pad is shown in Figure 13.

The design diagram included the following loads: the horizontal load P_h_ equal to 41.7 kN (load operation mode of the air distributor) and the vertical load P_ff_, i.e., the friction force equal to 20.85 kN.

The calculation was carried out according to the theory of maximum stresses. It is also known as the Coulomb criterion based on the theory of maximum stresses. According to this theory, fractures appear when the maximum stress reaches the tensile strength of the material for simple stretching. This criterion is used for brittle materials [33,34,35].

**Figure 14:**
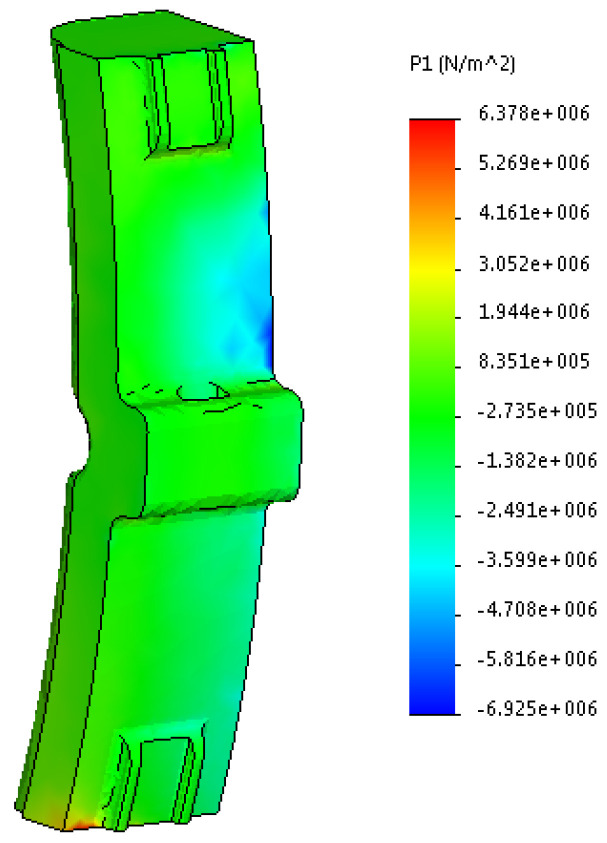
First main stress in the worn pad.

**Figure 15:**
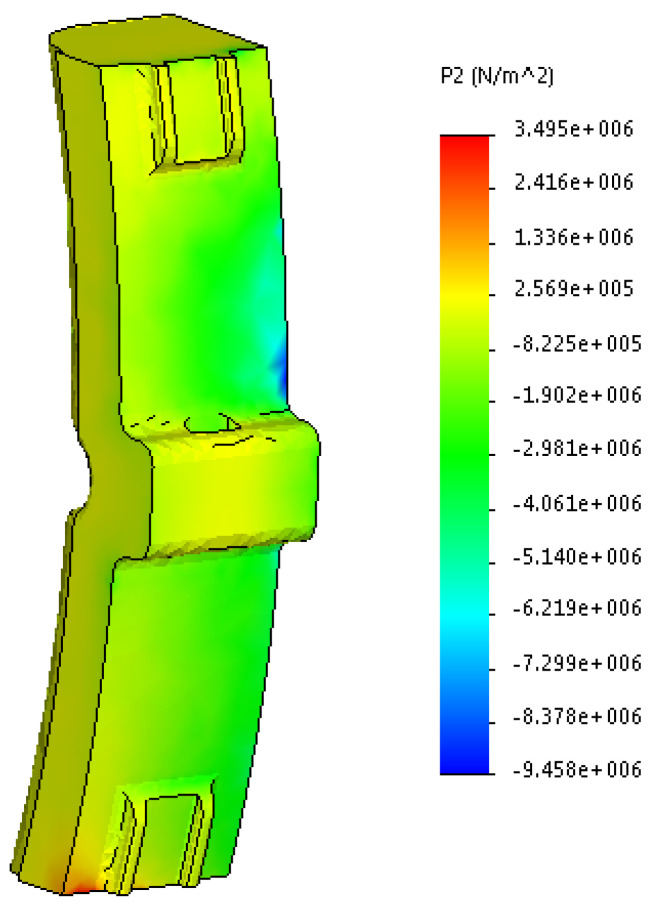
Second main stress in the worn pad.

**Figure 16:**
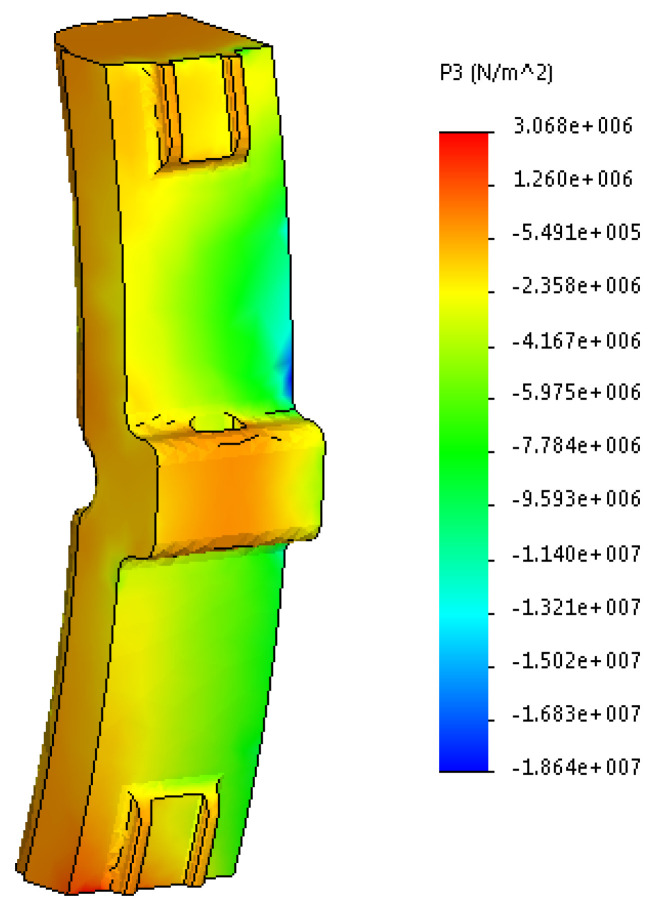
Third main stress in the worn pad.

The results are shown in Figure 14, Figure 15 and Figure 16.

By analyzing the obtained stress diagrams of the pad, it is possible to conclude that their maximum values are for main stress III and equal to about 19 MPa. Thus, the resulting stress value is 21% higher than the permissible value.

## 7. Discussion

Within the framework of studies of train traffic safety, the strength of the brake pad, including its uneven loading, has been investigated. This has been performed using a mathematical tool for determining the force factors that act on the pad presented as a rod system. The calculation has demonstrated that if the uneven loading is taken into account, the strength of the pad is not ensured. Moreover, the stresses in the pad are 29% higher than permissible.

The developed mathematical tool can be used to optimize the geometric parameters of the brake pad by the moment of resistance. These issues will be studied at further stages of the research.

In order to test the obtained results, the strength of the pad was determined using the finite element method implemented in SolidWorks Simulation. The Coulomb criterion, which is used for determining the strength of brittle materials, was taken as the calculation criterion. It has been found that the maximum stresses in the pad are about 19 MPa (Figure 16). They are 21% higher than permissible values and occur in the back of the pad. The discrepancy between the results of calculation when applying mathematical and computer modeling was about 9%.

The limitation of this study is that it is valid only for composite pads used for freight wagons operating on the 1520 mm track gauge.

The value of this study lies in the fact that the authors obtained the method for calculating brake pads under uneven loading. This result is very important because it will allow diagnostics of pads with unacceptable wear by creating special sensors. In turn, this will help improve train safety and, consequently, the efficiency of rail transportation.

A disadvantage of this study is that the proposed mathematical tool cannot produce the stress distribution fields in the pad since it is considered to be a rod system.

The advantage of the study in comparison with [2,3,4,5,6,7,8,9,10,11,12,13,14,15,16,17,18] is that the authors considered the uneven loading of the brake pad, which is typical in operation. However, this problem has not been given attention to so far.

A further stage in the field is the optimization of the geometric parameters of the brake pad. Also of importance are the issues of experimental determination of the strength of the brake pad under uneven loading; they will be considered in further research by the authors.

The results of this study will contribute to theoretical developments and recommendations aimed at improving the brake systems of freight wagons and traffic safety.

It is considered that future research in this field will be focused on performing real tests in order to find out the load of the investigated brake pads. Such tests require the implementation of tensometric sensors to the brake pad body in proper locations. It will be possible to detect the load distribution in the brake pad by means of the tensometric sensors and to verify the achieved results with the simulation computations. The intended tests will be performed on a special test stand at the authors’ workplace, which is equipped with corresponding sensors for the detection of needed outputs [36,37,38,39,40].

## 8. Conclusions

1. The causes of the uneven loading of the brake pad in operation have been identified. It has been found that one of the most important of them is a failure of the device for parallel retraction of brake shoes, which can result in constant contact of the upper part of the pad with the wheel during the train movement with brakes released. Also, the reason for the uneven loading of the pad is the deviation of the general center of gravity of the pad from the center of the hole in the brake strut.
2. The mathematical tool for determining the strength of the pad unevenly loaded has been proposed. It has been taken into account that the pad is a curvilinear beam on which external loads are acting in the form of concentrated forces and bending moment. In this case, classical approaches to the strength of materials and structural mechanics were used.
3. The results of the pad calculation at the values of the external vertical load *F* = 20.55 kN and the bending moment *M* = 68.8 kN∙m have proven that the stresses arising in it are 21.1 MPa. Therefore, they exceed the permissible values by 29%. This demonstrates that under the action of static forces applied unevenly across the area of the pad, the strength of the pad is not ensured.
4. The stress distribution fields in the brake composite pad of the wagon have been investigated, including the uneven loading. In this case, the finite element method was used, which is implemented in SolidWorks Simulation. The maximum stress criterion was used as a design criterion. The calculation was carried out for the loading operational mode of the air distributor with ref. No. 483-000. The stress state diagrams of the pad have been obtained. At the same time, the maximum stresses are typical for main stress III, and they amount to about 19 MPa. These stresses are 21% higher than permissible values and occur in the back of the pad.
5. The results of the study indicate that the uneven loading on the pad due to the stresses that exceed permissible ones can cause the destruction of the pad during braking, which can cause traffic accidents in operation. Therefore, it is necessary to implement scientific solutions during the design and creation of innovative brake systems for bogies that will eliminate the uneven loading in triangular systems, reduce the operating and maintenance costs of the wagon fleet, and guarantee train traffic safety.
6. The research will be useful for those who are concerned about designing innovative rolling stock units and improving the operational efficiency of railway transport.

## Figures and Tables

**Table 1:** The main characteristics of the material.

Indicator	Value
Brinell hardness, HB	1.2–3.0
Modulus of elasticity, Pa	5000
Poisson’s ratio	0.37
Mass density, kg/m^3^	2200
Compressive strength, MPa, not less than	15
Thermal expansion coefficient, K^−1^	4.1 × 10^−6^

## Data Availability

Data are contained within the article.
